# Supplementary material for: Deciphering Nicotine-Driven Oncogenesis in Head and Neck Cancer: Integrative Transcriptomics and Drug Repurposing Insights
Source: Cancers (Basel). 2025 Apr 24;17(9):1430. doi: 10.3390/cancers17091430 (PMC12070984; doi:10.3390/cancers17091430)
Supplement: Supplementary file 1 [file cancers-17-01430-s001.zip › Supplementary Table S1.pdf]

Supplementary Table S1. List of Nic-HNC gene set.

Gene Symbol	Entrez Gene	Gene Title	Microarray Nic/Pt GEOMEAN (log2)	TCGA-HNSC T/N FC (log2)
**<u>Up-regulation</u>**				
SPOCK1	6695	sparc/osteonectin, cwcv and kazal-like domains proteoglycan (testican) 1	1.87	1.15
TNFSF10	8743	tumor necrosis factor (ligand) superfamily, member 10	1.84	1.12
ZNF281	23528	zinc finger protein 281	1.74	1.37
SCD	6319	stearoyl-CoA desaturase (delta-9-desaturase)	1.73	1
FZD6	8323	frizzled class receptor 6	1.62	1.43
MDK	4192	midkine (neurite growth-promoting factor 2)	1.54	1.45
SPARC	6678	secreted protein, acidic, cysteine-rich (osteonectin)	1.54	2.61
BFAR	51283	bifunctional apoptosis regulator	1.47	1.02
SGPP1	81537	sphingosine-1-phosphate phosphatase 1	1.42	1.09
HLTF	6596	helicase-like transcription factor	1.41	1.39
CTSC	1075	cathepsin C	1.41	1.51
FKBP14	55033	FK506 binding protein 14	1.40	1.23
PTGS2	5743	prostaglandin-endoperoxide synthase 2 (prostaglandin G/H synthase and cyclooxygenase)	1.40	1.01
HACD3	51495	3-hydroxyacyl-CoA dehydratase 3	1.39	1.07
DDX60	55601	DEAD (Asp-Glu-Ala-Asp) box polypeptide 60	1.39	1.79
SPATS2	65244	spermatogenesis associated, serine-rich 2	1.37	1.27
SERPINE1	5054	serpin peptidase inhibitor, clade E (nexin, plasminogen activator inhibitor type 1), me	1.36	3.81
SERPINE2	5270	serpin peptidase inhibitor, clade E (nexin, plasminogen activator inhibitor type 1), me	1.36	1.43
ABCA1	19	ATP binding cassette subfamily A member 1	1.36	1.07
ITGA6	3655	integrin alpha 6	1.32	2.26
GALNT6	11226	polypeptide N-acetylgalactosaminyltransferase 6	1.30	2.63
RCN1	5954	reticulocalbin 1, EF-hand calcium binding domain	1.28	1.05
RPN1	6184	ribophorin I	1.27	1.02
SEL1L3	23231	sel-1 suppressor of lin-12-like 3 (C. elegans)	1.24	1.65
IFIT3	3437	interferon-induced protein with tetratricopeptide repeats 3	1.21	2.31
UBXN7	26043	UBX domain protein 7	1.20	1.18
SEMA3C	10512	sema domain, immunoglobulin domain (Ig), short basic domain, secreted, (semaphorin) 3C	1.20	1.61

IFI44	10561	interferon-induced protein 44	1.19	2.44
ADAM10	102	ADAM metallopeptidase domain 10	1.17	1.01
PRNP	5621	prion protein	1.11	1.22
SCPEP1	59342	serine carboxypeptidase 1	1.11	1.14
SKIL	6498	SKI-like proto-oncogene	1.10	1.19
NFE2L3	9603	nuclear factor, erythroid 2-like 3	1.10	1.51
IGFBP3	3486	insulin like growth factor binding protein 3	1.09	1.63
HEXB	3074	hexosaminidase B (beta polypeptide)	1.09	1.03
HSPA13	6782	heat shock protein 70kDa family, member 13	1.08	1.05
FXYD5	53827	FXYD domain containing ion transport regulator 5	1.07	1.56
MINPP1	9562	multiple inositol-polyphosphate phosphatase 1	1.07	1.33
IGFBP6	3489	insulin like growth factor binding protein 6	1.07	1.14
HLA-B	3106	major histocompatibility complex, class I, B	1.06	1.72
FNDC3B	64778	fibronectin type III domain containing 3B	1.05	2.06
EXT2	2132	exostosin glycosyltransferase 2	1.05	1.1
MMP13	4322	matrix metallopeptidase 13	1.04	4.35
PPT1	5538	palmitoyl-protein thioesterase 1	1.02	1.25
SLC39A14	23516	solute carrier family 39 (zinc transporter), member 14	0.99	1.48
B2M	567	beta-2-microglobulin	0.97	1.17
PON2	5445	paraoxonase 2	0.96	1.13
LRRC8D	55144	leucine rich repeat containing 8 family, member D	0.96	1.16
IL7R	3575	interleukin 7 receptor	0.96	1.78
SERPINH1	871	serpin peptidase inhibitor, clade H (heat shock protein 47), member 1, (collagen bindin	0.94	2.57
LPAR3	23566	lysophosphatidic acid receptor 3	0.94	1.32
LAPTM4B	55353	lysosomal protein transmembrane 4 beta	0.94	1.33
TGFBI	7045	transforming growth factor, beta-induced, 68kDa	0.93	3.63
PTPRK	5796	protein tyrosine phosphatase, receptor type, K	0.93	1.35
ST6GALNAC2	10610	ST6 (alpha-N-acetyl-neuraminyl-2,3-beta-galactosyl-1,3)-N-acetylgalactosaminide alpha-2	0.90	1.01
LSR	51599	lipolysis stimulated lipoprotein receptor	0.90	1.08
MX1	4599	MX dynamin-like GTPase 1	0.90	1.44
ARL14	80117	ADP-ribosylation factor like GTPase 14	0.90	1.16
LRP12	29967	LDL receptor related protein 12	0.88	1.74
HERC5	51191	HECT and RLD domain containing E3 ubiquitin protein ligase 5	0.88	2.23
GGH	8836	gamma-glutamyl hydrolase (conjugase, folylpolygammaglutamyl	0.87	1.2

		hydrolase)		
CAPRIN2	65981	caprin family member 2	0.86	1.18
HLA-C	3107	major histocompatibility complex, class I, C	0.84	1.34
ANO1	55107	anoctamin 1, calcium activated chloride channel	0.83	1.87
HLA-F	3134	major histocompatibility complex, class I, F	0.83	1.75
NEMP1	23306	nuclear envelope integral membrane protein 1	0.82	1.58
STK17A	9263	serine/threonine kinase 17a	0.81	1.25
INHBA	3624	inhibin beta A	0.81	4.69
GJA1	2697	gap junction protein alpha 1	0.79	1.69
OLFML2A	169611	olfactomedin like 2A	0.79	1.57
GRINA	2907	glutamate receptor, ionotropic, N-methyl D-aspartate-associated protein 1 (glutamate bi	0.79	1.03
P4HA2	8974	prolyl 4-hydroxylase, alpha polypeptide II	0.78	1.62
CHST11	50515	carbohydrate (chondroitin 4) sulfotransferase 11	0.78	2.29
PLPP2	8612	phospholipid phosphatase 2	0.77	1.16
HLA-G	3135	major histocompatibility complex, class I, G	0.76	1.65
IGF1R	3480	insulin-like growth factor 1 receptor	0.74	1.11
ATP2C1	27032	ATPase, Ca++ transporting, type 2C, member 1	0.74	1.2
TGFBR1	7046	transforming growth factor, beta receptor 1	0.73	1
JADE2	23338	jade family PHD finger 2	0.72	1.2
PRIM2	5558	primase, DNA, polypeptide 2 (58kDa)	0.71	1.54
PLAUR	5329	plasminogen activator, urokinase receptor	0.70	2.14
EMP3	2014	epithelial membrane protein 3	0.70	1.68
FADS1	3992	fatty acid desaturase 1	0.70	2.82
TUSC3	7991	tumor suppressor candidate 3	0.70	1.36
ATP1B3	483	ATPase, Na+/K+ transporting, beta 3 polypeptide	0.68	1.23
LPCAT1	79888	lysophosphatidylcholine acyltransferase 1	0.67	1.95
GABRE	2564	gamma-aminobutyric acid (GABA) A receptor, epsilon	0.67	1.59
EXTL2	2135	exostosin-like glycosyltransferase 2	0.66	1.3
DSG2	1829	desmoglein 2	0.66	2.19
P3H2	55214	prolyl 3-hydroxylase 2	0.65	2.38
CNTN1	1272	contactin 1	0.65	1.23
PLOD2	5352	procollagen-lysine, 2-oxoglutarate 5-dioxygenase 2	0.65	1.7
F2R	2149	coagulation factor II (thrombin) receptor	0.63	1.81
PROCR	10544	protein C receptor, endothelial	0.62	2.08
ADPGK	83440	ADP-dependent glucokinase	0.62	1.09
SQLE	6713	squalene epoxidase	0.60	1.32
ACVR1	90	activin A receptor type I	0.60	1.4
SKAP2	8935	src kinase associated phosphoprotein 2	0.60	1.06

MLF1	4291	myeloid leukemia factor 1	0.59	1.61
CASK	8573	calcium/calmodulin-dependent serine protein kinase (MAGUK family)	0.59	1.19
FOXD1	2297	forkhead box D1	0.58	1.75
SLC16A1	6566	solute carrier family 16 (monocarboxylate transporter), member 1	0.58	1.56
DLGAP5	9787	discs, large (Drosophila) homolog-associated protein 5	0.58	1.68
ITGA3	3675	integrin alpha 3	0.57	1.78
PTHLH	5744	parathyroid hormone-like hormone	0.56	4.4
PRSS23	11098	protease, serine, 23	0.55	1.4
TRAM2	9697	translocation associated membrane protein 2	0.55	2.02
PLOD3	8985	procollagen-lysine, 2-oxoglutarate 5-dioxygenase 3	0.55	2.26
TP63	8626	tumor protein p63	0.54	1.24
KIF20A	10112	kinesin family member 20A	0.54	1.7
KIF4A	24137	kinesin family member 4A	0.52	1.92
LAMB3	3914	laminin, beta 3	0.51	1.98
BTN3A2	11118	butyrophilin, subfamily 3, member A2	0.51	1.05
LAMC2	3918	laminin, gamma 2	0.51	4.06
STXBP1	6812	syntaxin binding protein 1	0.50	1.02
ENTPD7	57089	ectonucleoside triphosphate diphosphohydrolase 7	0.50	1.12
PDPN	10630	podoplanin	0.50	2.48
FXYD3	5349	FXYD domain containing ion transport regulator 3	0.49	1.67
DDX58	23586	DEAD (Asp-Glu-Ala-Asp) box polypeptide 58	0.49	1.41
DENND5A	23258	DENN/MADD domain containing 5A	0.49	1.15
LIMA1	51474	LIM domain and actin binding 1	0.48	1.19
ISG15	9636	ISG15 ubiquitin-like modifier	0.47	3.9
BAG2	9532	BCL2-associated athanogene 2	0.46	1.58
NETO2	81831	neuropilin (NRP) and tolloid (TLL)-like 2	0.46	2.3
TAP1	6890	transporter 1, ATP-binding cassette, sub-family B (MDR/TAP)	0.46	1.71
MYO10	4651	myosin X	0.44	1.55
DDIT4	54541	DNA damage inducible transcript 4	0.44	1.39
TLR2	7097	toll-like receptor 2	0.43	1.13
TOPBP1	11073	topoisomerase (DNA) II binding protein 1	0.42	1.15
KIF11	3832	kinesin family member 11	0.42	1.13
LARP6	55323	La ribonucleoprotein domain family, member 6	0.41	1.25
NEK2	4751	NIMA-related kinase 2	0.39	1.71
CHST7	56548	carbohydrate (N-acetylglucosamine 6-O) sulfotransferase 7	0.39	1.71
DHCR7	1717	7-dehydrocholesterol reductase	0.39	1.07
HSPA2	3306	heat shock 70kDa protein 2	0.38	1.03

LMAN2L	81562	lectin, mannose-binding 2-like	0.38	1.04
STEAP1	26872	six transmembrane epithelial antigen of the prostate 1	0.37	1.01
MMP2	4313	matrix metallopeptidase 2	0.36	1.86
GLIPR1	11010	GLI pathogenesis-related 1	0.34	1.51
GBP1	2633	guanylate binding protein 1, interferon-inducible	0.33	1.2
CLIC4	25932	chloride intracellular channel 4	0.29	1.73
VEGFA	7422	vascular endothelial growth factor A	0.23	1.16
KPNA2	3838	karyopherin alpha 2 (RAG cohort 1, importin alpha 1)	0.22	1.45
MICA	100507436	MHC class I polypeptide-related sequence A	0.18	1.07
FOXF2	2295	forkhead box F2	0.16	2.17
EPB41L4B	54566	erythrocyte membrane protein band 4.1 like 4B	0.12	1.77
IFIT1	3434	interferon-induced protein with tetratricopeptide repeats 1	0.10	1.93
IL1A	3552	interleukin 1 alpha	-0.04	2.05
DDIT3	1649	DNA-damage-inducible transcript 3	-0.55	1.19
**Down-regulation**				
ATP6V0A4	50617	ATPase, H+ transporting, lysosomal V0 subunit a4	-3.99	-0.57
MYL3	4634	myosin, light chain 3, alkali; ventricular, skeletal, slow	-2.98	-0.41
DUSP13	51207	dual specificity phosphatase 13	-2.39	-0.55
SGCA	6442	sarcoglycan alpha	-2.34	-0.61
CLIC3	9022	chloride intracellular channel 3	-2.31	0.11
CSTA	1475	cystatin A (stefin A)	-2.09	-0.44
FHL1	2273	four and a half LIM domains 1	-1.78	-0.61
HMGCS2	3158	3-hydroxy-3-methylglutaryl-CoA synthase 2 (mitochondrial)	-1.59	-0.75
FOS	2353	FBJ murine osteosarcoma viral oncogene homolog	-1.56	-1.50
DKK4	27121	dickkopf WNT signaling pathway inhibitor 4	-1.42	-0.49
SERPINB1	1992	serpin peptidase inhibitor, clade B (ovalbumin), member 1	-1.39	-0.88
DUSP1	1843	dual specificity phosphatase 1	-1.37	-0.87
EGR1	1958	early growth response 1	-1.34	-1.13
SLC25A23	79085	solute carrier family 25 (mitochondrial carrier; phosphate carrier), member 23	-1.26	-0.51
SRPK3	26576	SRSF protein kinase 3	-1.24	-0.63
EPHB6	2051	EPH receptor B6	-1.20	-0.68
MUC1	4582	mucin 1, cell surface associated	-1.10	-0.90
LGALSL	29094	lectin, galactoside-binding-like	-1.05	-0.49
RAB17	64284	RAB17, member RAS oncogene family	-1.04	-0.49
